# Zinc Treatment to Under-five Children: Applications to Improve Child Survival and Reduce Burden of Disease

**DOI:** 10.3329/jhpn.v26i3.1901

**Published:** 2008-09

**Authors:** Charles P. Larson, S.K. Roy, Azharul Islam Khan, Ahmed Shafiqur Rahman, Firdausi Qadri

**Affiliations:** ICDDR, B, Mohakhali, Dhaka 1212, Bangladesh

**Keywords:** Child survival, Diarrhoea, Acute, Diarrhoea, Infantile, Interventions, Morbidity, Zinc, Zinc deficiency, Zinc therapy, Bangladesh

## Abstract

Zinc is an essential micronutrient associated with over 300 biological functions. Marginal zinc deficiency states are common among children living in poverty and exposed to diets either low in zinc or high in phytates that compromise zinc uptake. These children are at increased risk of morbidity due to infectious diseases, including diarrhoea and respiratory infection. Children aged less than five years (under-five children) and those exposed to zinc-deficient diets will benefit from either daily supplementation of zinc or a 10 to 14-day course of zinc treatment for an episode of acute diarrhoea. This includes less severe illness and a reduced likelihood of repeat episodes of diarrhoea. Given these findings, the World Health Organization/United Nations Children's Fund now recommend that all children with an acute diarrhoeal illness be treated with zinc, regardless of aetiology. ICDDR.B scientists have led the way in identifying the benefits of zinc. Now, in partnership with the Ministry of Health and Family Welfare, Government of Bangladesh and the private sector, the first national scaling up of zinc treatment has been carried out. Important challenges remain in terms of reaching the poorest families and those living in remote areas of Bangladesh.

## INTRODUCTION

It is now over a decade since the publication of the landmark articles by Sazawal *et al.* and Roy *et al.* which demonstrated the efficacy of orally-administered zinc in the treatment of acute childhood diarrhoea (1,2). Since then, several randomized hospital- and community-based trials have consistently demonstrated the efficacy of zinc treatment for acute or persistent diarrhoea in children aged less than five years (under-five children) (3-6). Pooled analyses of published data demonstrate that zinc reduces the duration and severity of acute diarrhoea and the likelihood of a prolonged episode (7,8). Results from these efficacy trials were then replicated by a community-based, effectiveness trial of zinc treatment for acute childhood diarrhoea carried out in the ICDDR, B rural field site in Matlab. In this trial in which children received daily zinc treatment for each episode of diarrhoea, children in the zinc intervention group had a shorter duration of illness, a reduced likelihood of a repeat episode of diarrhoea, and non-injury mortality. The reduction in mortality was very substantial (50%) (9). This study and several more-cited investigations were carried out by scientists at ICDDR, B who continue to study the effects of zinc on diarrhoeal and other illnesses, most notably childhood pneumonia.

The World Health Organization (WHO) has estimated the global annual burden of mortality attributable to zinc deficiency to be 750,000 deaths (10). It is anticipated that over one-half of these deaths could be averted through the successful application of zinc as a treatment for childhood diarrhoea (11). Given this potential reduction in mortality and the strength of the evidence at hand in support of zinc treatment, the WHO/United Nations Children's Fund (UNICEF) issued, in May 2004, a joint statement on updated guidelines for the management of childhood diarrhoea (12). This includes the recommendation that all under-five children be treated with zinc (20 mg/day if age is 6-59 months and 10 mg/day if age is less than six months) for 10-14 days. This recommendation is now a policy of the Ministry of Health and Family Welfare, Government of Bangladesh, with a slight modification to include children starting at two months of age.

- Zinc deficiency is estimated to be related to 750,000 deaths annually.
- WHO and UNICEF now recommend 10-14 days of zinc treatment for under-five children with each diarrhoea illness.
- Zinc treatment is inexpensive, safe, and easy.
- Zinc treatment shortens the diarrhoea episode, reduces the risk of episode being persistent, and reduces the risk of future diarrhoea or pneumonia.
- Zinc treatment reduces overall mortality.
- Now the task is to provide zinc treatment to every child in Bangladesh with each episode of diarrhoea.

This paper summarizes our understanding of zinc deficiency in children, its relationship with childhood morbidity and mortality, the strategies that have been tested to supplement zinc, and the bene-fits of these interventions. This is followed by a discussion of future research priorities and their applicability to health policy and planning.

## ZINC DEFICIENCY IN EARLY CHILDHOOD

Population-based estimates of the occurrence of zinc deficiency in young children are hindered by the lack of an accurate measure of zinc status. Current estimates are based upon one or a combination of zinc-deficiency indicator(s). These include rates of stunting, the amount of zinc in national food supplies, serum zinc levels, and histories of dietary intake.

Despite the limitations in accurately estimating zinc levels, it is now recognized that mild-to-moderate zinc deficiency due to inadequate dietary intake is prevalent in all parts of the world. The higher prevalence of zinc deficiency in developing countries is due primarily to low intake of zinc from animal sources, high dietary phytate content (that limits the bioavailability of zinc), and inadequate food intake (13). A population-level analysis from national food-balance sheets has estimated that 21% of the world population is at risk of inadequate zinc intake; however, the percentages are much higher in least-developed countries (14,15). These children are especially prone to zinc deficiency because of poor dietary quality and increased faecal loss of zinc due to repeated gastrointestinal infections (8). Children with modest levels of chronic zinc deficiency do not manifest any observable clinical signs that would alert clinicians to its presence, thus making it a hidden disorder. Bangladesh has one of the highest prevalence levels in the world, affecting over 50% of all under-five children (15).

Being a micronutrient component in many metallo-enzymes and poly-ribosomes involved in cellular function, zinc supports normal growth and development during pregnancy, childhood, and adolescence (16). It is essential for metabolism, cellular growth, and immune function (17). Despite its essential role, overt clinical syndromes associated with zinc deficiency in humans are rare.

The first published description of clinically-evident zinc deficiency due to nutritional causes in otherwise normal humans was documented in the Middle East in the 1960s among adolescent boys, characterized by stunted growth and delayed sexual maturation that were reversible with supplementation of zinc (18). One well-known zinc-deficiency disorder with overt clinical signs is acrodermatitis enteropathica, a genetic autosomal recessive disease with an inborn defect in metabolism that results in reduced intestinal absorption of zinc (19). The discovery of this genetic disorder and its rapid resolution when treated with zinc alerted clinicians to the potential impact of zinc as a clinical deficiency disorder in humans. Not long after this discovery, zinc deficiency was also found to occur in adult patients on total parenteral nutrition, which was attributable to the failure to add zinc in the intravenous infusates (20,21). These individuals suffered from loss of memory, skin disorders, loss of taste, and increased susceptibility to infection—all of which disappeared when zinc was added.

It is now well-known that mild-to-moderate zinc deficiency due to inadequate dietary intake is pre-valent in all parts of the world, with higher pre-valence in developing countries, due primarily to low intake of zinc from animal sources, high dietary phytate content, and inadequate food intake (13).

## ZINC, INFECTION, AND IMMUNITY

Closely linked to these effects is the important role it plays in maintaining normal immune function. Results of studies have suggested that zinc deficiency impairs cell regeneration, epithelial barrier functions, and linear growth (22). Zinc deficiency also impairs immunocompetence with reduced cell-mediated immune responses, decreased T-lymphocytes, abnormal T-helper and/or suppressor functions, impaired macrophage function, and reduced killer cells and antibody-dependent cytotoxicity (23,24). The levels of complement in blood increases with supplementation of zinc in children with acute diarrhoea (Qadri F. Personal communication, 2006). Zinc levels also modulate the function of monocytes, macrophages, and neutrophils polymorphs and in the release of reactive free radicals from phagocytes (23). These impairments in immune function occur even with modest levels of zinc deficiency.

The possible mechanisms of the effect of zinc treatment on the duration and severity of diarrhoea include improved absorption of water and electrolytes by the intestines (25-27), regeneration of gut epithelium (28-31), increased levels of enterocyte brush-border enzymes (32,33), and enhanced immunologic mechanisms for the clearance of infection. Supplementation of zinc improves immunity (34-36) and may, hence, promote rapid clearance of diarrhoeal pathogens from the intestine.

Innate immunity is the body's first line of defence to pathogens, and its functions are also disturbed by altered zinc levels. Natural killer cell numbers and function are dependent on normal levels of serum zinc (37). These levels also modulate the function of monocytes, macrophages, and neutrophils (23). Zinc is also required for the development and activation of T-lymphocytes. When zinc supplements are given to individuals having low levels of zinc, the numbers of T-cell lymphocytes circulating in the blood increase, and the ability of lymphocytes to fight against infection improves.

## ZINC DEFICIENCY AND MORBIDITY

Given the numerous biologic functions dependent upon normal levels of zinc, particularly immunity, it is not surprising to find that zinc deficiency is associated with numerous infectious illnesses, but the link between diarrhoea and zinc is especially well-established. Diarrhoea leads to loss of zinc and abnormalities of zinc metabolism. Substantial amounts of zinc are lost during acute diarrhoea: daily losses of zinc during diarrhoea can be as high as 160 µg/kg per day in children (38).

How important are marginal deficiencies in zinc? Clinical and field studies have consistently observed an association between zinc deficiency and morbidity due to infectious diseases, particularly diarrhoea in early childhood (39-41). Marginal zinc deficiency is associated with about a 50% increased risk and number of days with diarrhoea. However, zinc deficiency results in higher rates of other infectious diseases as well, including skin infections, respiratory infections, malaria, and delayed wound healing (17). Overall, zinc-deficient children are at a three-fold increased risk of an acute respiratory infection (40,41).

## EFFECTS OF ZINC SUPPLEMENTATION

The recommended dietary allowance (RDA) for infants aged 0-6 month(s) is 2.0 mg, and it is 3.0 mg per day for young children aged 7-36 months (42). However, the amount of zinc needed in young infants to maintain a positive zinc balance in areas with a high prevalence of zinc deficiency is unknown. The majority of published results of efficacy trials of zinc treatment have tested doses ranging from 10 mg (infants) to 20 mg (under-five children) of elemental zinc per day, a dosage that is safe in these children. Doses of up to 70 mg twice a week have been provided without an any toxic effect or clinically-significant copper deficiency (43).

Several controlled zinc-treatment trials over the past decade have demonstrated the beneficial role of zinc in the prevention and treatment of diarrhoea (1-3,6-9,44,45). Meta-analyses of these trials estimate that children, aged three months to five years, who receive zinc for the treatment of a diarrhoeal illness (20 mg/day for 10 days), recover faster and experience a 30% reduction in the likelihood of developing prolonged diarrhoea. Over 3-6 months following treatment, there is a 30% decreased likelihood of a subsequent episode and an estimated 50% reduction in non-injury mortality (8,9,46,47).

Zinc has also been demonstrated to be effective as a daily supplement in the prevention of diarrhoeal illnesses. A community-based, double-blind, randomized trial in India observed a 26% lower incidence of diarrhoea and a 35% lower prevalence in children who received daily supplementation of zinc for six months (48). A trial of zinc supplementation (10 mg/day) in growth-retarded Vietnamese children observed a 71% lower incidence of diarrhoea (49). A similar trial in Mexico found a 36% lower incidence of diarrhoeal episodes in zinc-supplemented (20 mg/day) children (50). It has also been observed that supplementation of zinc reduces the incidence of persistent diarrhoea in zinc-deficient children and reduces the risk of dysentery (51). A report from Guatemala also indicated that supplementation of zinc reduces the incidence of all types of diarrhoea (52). Studies in Bangladesh also demonstrated that supplementation of zinc to children with diarrhoea improves their growth (53).

The table provides a summary of several recently-published studies examining the preventive effects of zinc provided either as a treatment or as a daily supplement. The table shows that either strategy appears to provide protection against the future occurrence of acute diarrhoea, and the protection lasts for 3-6 months following treatment or cessation of supplementation. The treatment also decreases the likelihood that an acute episode of diarrhoea will progress on to a prolonged (>7 days) or persistent (>14 days) episode of diarrhoea.

**Table.** Summary of studies testing for preventive impact of zinc as a treatment or supplement

Study	Setting	Zinc dose	Results (reduction effect)
Roy SK, 1999 (53)	Bangladesh, hospital-based	20 mg/day × 14 days of treatment	14% ACD 58% ALRI
Baqui AH, 2002 (9)	Bangladesh, community-based	20 mg/day × 14 days of treatment	15% ACD 19% ACD hospital, 51% non-injury mortality
Sazawal S, 1996 (51)	India	10 mg/day supplement × 6 months	21% PCD 14% ACD
Rosado JL, 1997 (50)	Mexico	20 mg/day supplement × 12 months	20% ACD
Ruel MT, 1997 (52)	Guatemala	10 mg/day supplement × 7 months	22% ACD 67% PCD
Ruz M, 1997 (54)	Chile	10 mg/day supplement × 14 months	No effect on morbidity
Sazawal S, 1997 (48)	India	10 mg/day supplement × 6 months	17% in ACD (effect limi-ted to subjects aged >12 months)
Sazawal S, 1998 (55)	India	10 mg/day supplement × 17 weeks	45% ALRI
Bhandari N, 2002 (56,57)	India	10-20 mg/day supplement × 4 months	12% ACD 31% PCD 25% ALRI

ACD=Acute childhood diarrhoea; ALRI=Acute lower respiratory infection; PCD=Persistent childhood diarrhoea

## ZINC SAFETY

Acute zinc toxicity due to excess administration (225-450 mg of zinc) includes gastrointestinal symptoms, such as nausea, vomiting, epigastric pain, abdominal cramps, and bloody diarrhoea (58). Recent randomized clinical trials among adults and adolescents with acne, anorexia nervosa, macular degeneration, common cold, and tuberculosis, using elemental zinc in the dose range of 50-100 mg/day, frequently reported nausea and vomiting as side-effects (59-61).

Transient vomiting or nausea among adults at doses of 50 mg/day or higher are well-known side-effects of zinc (62,63). Whether similar effects at the lower dose of 10-20 mg/day will be observed in children is unclear. Strand reported a nearly two-fold increase in vomiting when treating Nepalese children with 15-30 mg/day (6). Whether this was due to a direct side-effect of zinc or due to inadequate masking of the metallic taste of zinc could not be differentiated. Other trials, including the effectiveness trial of Baqui *et al*., did not report increased risks of vomiting (9). In none of the cited studies was the increased risk of vomiting linked to adverse serious clinical outcomes.

To document if zinc does increase vomiting in children acutely ill with gastrointestinal illness, a randomized, double-blind, placebo-controlled trial was carried out in the short-stay unit of the ICDDR, B hospital and an adjacent outpatient clinic (64). Many children had vomiting as part of their illness, but the concern was that zinc might increase the rate and/or severity of vomiting. Results of this study showed that children who received the dispersible zinc tablet formulation in a dose of 20 mg had a 14% increased risk of vomiting attributable to zinc following the first treatment dose. This is equivalent to one additional vomiting episode for every seventh child treated. The vomiting does not occur immediately after administration, but about 10 minutes later and is transient in nature, occurring only once in over 90% of children. Thus, it does seem that treatment with zinc does increase the rate of vomiting somewhat in children who are acutely ill with diarrhoea, but the increase is slight and transient. Vomiting has not been a problem in children who are not acutely ill with gastrointestinal diseases.

## ZINC ADMINISTRATION ALONE OR IN COMBINATION WITH OTHER MICRONUTRIENTS

The clear benefits have raised questions about the most appropriate use of zinc. Should it be used as a single therapy, or should it be combined with other minerals and vitamins, especially when used in a dietary supplement or a food fortificant? Answering these questions will require additional research to validate the most effective products for children, especially for long-term use. However, for use in treating children with diarrhoea, the safety and benefits of zinc alone are clearly defined, and recommendations for its use are compelling. We, thus, feel that programmes to implement the re-commendations of WHO/UNICEF need to be scaled up as rapidly as possible, even while research continues to identify the best products for dietary supplementation or fortification. It should be noted that recent efforts to combine zinc with iron/folic acid in East Africa, an area with high rates of malaria, found higher rates of hospitalization and mortality in children who received the combined supplement (65). Such combinations may, thus, need to be examined more closely to assure freedom from adverse events.

## SCALING UP ZINC AS A TREATMENT FOR CHILDHOOD DIARRHOEA

The greatest challenge facing health researchers, practitioners, and funding agencies is how to translate the proven effectiveness of zinc as a treatment into action that will benefit the lives of young children, particularly those living in conditions of chronic poverty and malnutrition. In 2003, ICDDR, B launched the Scaling Up Zinc for Young Children (SUZY) Project with the aim of setting Bangladesh on the path to providing all under-five children with diarrhoea with zinc treatment, irres-pective of gender, income, or geographic location. To attain this goal, the SUZY Project has been organized around five key activities: (a) registration, production, and distribution of zinc tablet, (b) promotion among healthcare providers and mass media campaign, (c) training of professionals and introduction of zinc treatment into public, private and NGO delivery systems, (d) formative and operations research in support of scaling up, and (e) knowledge transfer from Project findings.

With regard to registration and production, the Project decided to pursue the scaling up of a dispersible, 20-mg zinc sulphate tablet (which is now known as ‘Baby Zinc’ in Bangladesh), as recommended by WHO. The tablet is placed in a spoon or a small cup and water added which leads it to disperse into a sweet, vanilla-flavoured syrup that masks the taste of zinc. The treatment is packaged in a 10-tablet blister pack, and caretakers are instructed to give one tablet per day for 10 days. It was not known at the time of launching whether young Bangladeshi children would find the tablet formulation acceptable, whether caretakers could correctly adhere to treatment instructions. This was studied in rural and urban settings, with findings indicating that the formulation was highly acceptable and that treatment instructions were easily followed. Ninety-eight percent of caretakers prepared the syrup correctly; over 90% perceived that their children found the taste to be acceptable, and the tablets were given, on average, for eight days (66). There were also concerns regarding side-effects associated with the formulation. As already described, it was found that the formulation is associated with a transient increase in the risk of vomiting, but with no adverse clinical consequences (64).

Formative studies were undertaken that involved caretakers of children with an active case of diarrhoea, healthcare providers (licensed and unlicensed), drug vendors, and medical representatives (drug salesmen). A recent baseline survey conducted throughout Bangladesh showed that most (>90%) consultations of healthcare providers for a childhood diarrhoea episode involve the private sector (67). This survey also confirmed the disparities in care received, favouring households with higher wealth, and those living in urban settings. This has led the SUZY Project to develop a promotion strategy that emphasizes awareness-building in the private sector, but also sensitization and training programmes for the government (public) and NGO sectors. The formative caretaker and provider interviews have led to the development of a frequently-asked databank. Some more frequently-asked questions and responses are found in the Appendix.

## CURRENT ISSUES AND FUTURE RESEARCH PRIORITIES

### Healthcare-delivery systems

To reach all children with diarrhoea, zinc treatment will need to be introduced and sustained within the public, private and NGO service-delivery systems. Each of these systems has its unique set of strengths and weaknesses that must be taken into account in the planning stages. Unanswered questions include whether or not zinc treatment can be introduced through community health workers or depot-holders, the impact of over-the-counter availability of zinc on the use of health services, and the misuse of zinc for untested disorders, such as acute respiratory tract infections, poor growth, and loss of appetite.

### Financing

As a preventive measure, treatment of childhood diarrhoea with zinc has been estimated to be one of the most cost-effective interventions available. Nonetheless, because of the shear frequency of childhood diarrhoea, the costs either at the household level or those assumed by the public or private sector could be substantial. It remains to be demonstrated what the longer-term impact of a successful scaling-up campaign will be. It is reasonable to assume that an initial investment in zinc treatment will eventually lead to deceased expenditure on other drugs, particularly antibiotics, and the costs avoided by preventing episodes of future illness. Until these assumptions are verified and appropriate information is disseminated, decision-makers will be reticent to commit public or other subsidized financial resources, e.g. NGO clinics. A further constraint faced by the private sector, particularly the pharmaceutical industry, is the current lack of sound data upon which to estimate demand and pricing of product.

### Combining zinc and iron

Iron and zinc deficiencies commonly coincide in early childhood. The obvious conclusion is to treat both the conditions simultaneously. It is not yet clear whether or not this combined approach should be made a public-health policy; in fact, there is now concern about using iron routinely in malaria-endemic areas. It is known that, in children receiving zinc therapy, levels of serum iron are adversely affected. Evidence is also emerging that, while children are receiving iron supplementation, the effects of zinc supplementation in terms of reduced morbidity are cancelled out—at least in areas with high rates of malaria. It will, therefore, be important to test alternative supplementation and zinc-treatment strategies and confirm these results in the desired beneficial effects prior to establishing policies in favour of combined supplementation. Finally, it may be the case that combined therapies will have a differential impact based upon the nutritional status of a child and the severity of his/her micronutrient status. This requires further study.

Impact on diarrhoea-management practices and use of drugs: As zinc treatment is introduced, what will happen to existing diarrhoea-management practices? Will zinc be added to existing treatments, such as ORS (desired) and antibiotics (not desired)? Will providers and drug vendors view zinc as an opportunity or as a threat, and for what reasons? Given the first national scaling up of zinc is occurring in Bangladesh, it is difficult to predict how this will influence current practices, thus the importance of having in place the capacity to monitor for the potential desired and undesired changes in management practices.

### Home management of childhood diarrhoea

Caretakers in Bangladesh lead the world in the use of ORS. We need to build upon this success as zinc treatment in childhood diarrhoea is introduced through mass media and promotion. Given that zinc will be available over-the-counter in stores without prescription, caretakers will have easy access to it. The challenge will be to develop and confirm the effectiveness of public education that aims at improving home-management practices.

## SUMMARY

Zinc is one of the most important new health interventions which is only now beginning to be scaled up in Bangladesh and will, hopefully, be soon introduced in other countries. It has the potential to be one of the most cost-effective health interventions for child survival as were ORS and measles vaccine. When studies first started, it was thought that it might be a way of improving treatment of diarrhoea somewhat, but it has turned out to be a real life-saver.

## Appendix

**Frequently-asked Questions about Use of Zinc in Treating Children with Diarrhoea**

1. What is zinc? What does it do? Zinc is a mineral, not a vitamin. It is an essential micronutrient found in almost every cell in the body. It stimulates the activity of approximately 100 enzymes that help in multiple biological functions of the human body. Importantly, it supports a healthy immune system and is needed for wound healing.
2. What are the natural sources of zinc? The natural sources of zinc are: red meat, poultry, beans, nuts, whole grain, dairy products, and certain sea-foods, such as oysters.
3. Can the zinc tablet be given to a child without consulting a doctor? Yes, it is not necessary to consult a doctor to give zinc. Zinc should be given to any child with diarrhoea regardless of the type of diarrhoea. For children who exhibit severe symptoms, such as vomiting or dehydration, it is important to consult a healthcare provider first.
4. What is the dose and duration of zinc treatment in diarrhoea? Is there any side-effect of the above dose? The treatment of diarrhoea involves a 20-mg tablet once per day for 10 days. The 20-mg dose is perfect for the children aged from 6 months to <5 years. The only potential side-effect is transient vomiting.
5. Why tablets and not syrup? There are several zinc syrups available in the market, and these are as effective as the tablets during diarrhoea. The tablets are preferred because:
  - These are less costly
  - It is easier to give the correct dose and keep track of the number of days given
  - Tablets are easier to distribute and store
6. Can zinc be given by mixing it with juice, ORS, breastmilk, or any other liquids? The zinc tablet is meant to be dissolved in water. However, a spoonful of ORS or breast milk can replace a spoonful of water. Other fluids are not recommended.
7. Should a child be given another course of zinc treatment if he/she experiences a second episode of diarrhoea? Yes, all episodes of diarrhoea should be treated with zinc. Even if a child has recently completed a full course of zinc treatment, it is still safe to give a second dose.
8. Will zinc work for children above five years of age as well as it does for children aged below five years? Probably, it would. However, there is no scientific evidence demonstrating the effectiveness of zinc as a treatment for diarrhoea in children aged over five years. In addition, older children are less susceptible to the more severe-effects of diarrhoea, so they may not benefit to the same degree.
9. Can diarrhoea be treated with zinc only? Acute childhood diarrhoea should be treated with ORS AND zinc. In children with bloody diarrhoea or suspected cholera, an antibiotic may also be required. Zinc is not an alternative for ORS.
10. Can zinc be given to an exclusively-breastfed child? Yes, to treat diarrhoea, zinc can be given to an exclusively-breastfed child.
